# The use of transcranial direct current stimulation to facilitate motor skill reactivations of a choice reaction time task in adults

**DOI:** 10.14814/phy2.70630

**Published:** 2025-10-27

**Authors:** Michaela A. Wilson, Brach Poston, Zachary A. Riley

**Affiliations:** ^1^ Department of Kinesiology, School of Health and Human Sciences Indiana University‐Indianapolis Indianapolis Indiana USA; ^2^ Program in Health and Human Performance University of Pikeville Pikeville Kentucky USA; ^3^ Department of Kinesiology and Nutrition Sciences University of Nevada‐Las Vegas Las Vegas Nevada USA; ^4^ Interdisciplinary Ph.D. Program in Neuroscience University of Nevada‐Las Vegas Las Vegas Nevada USA

**Keywords:** motor skill, reaction time, reactivations, tDCS

## Abstract

Motor skill acquisition involves fast and slow learning phases, typically requiring extended practice. This study explored whether brief reactivations of a choice reaction time (CRT) task, combined with anodal transcranial direct current stimulation (tDCS) over the primary motor cortex (M1), could yield performance improvements comparable to full‐length practice. A total of 120 healthy adults were randomized into six groups varying in tDCS use (2 mA for 5 or 20 min) and practice duration (5 or 20 min). Reaction time (RT) and error rate were assessed across sessions. Across all groups, RT significantly improved from 423.5 ± 116.8 ms at pre‐test to 357.9 ± 63.4 ms post‐test (*p* < 0.001), of the first session. RTs at session 4 (377.8 ± 66.2 ms) remained significantly faster than baseline (*p* < 0.001), though slightly slower than immediate post‐test in session 1 (*p* = 0.172). No significant between‐group differences emerged in RT or error rate, though the brief reactivation + tDCS group achieved RTs similar to the full‐practice group. When separated by sex, women showed slower reaction times initially and less improvement in reaction times with practice. These results suggest that brief, tDCS‐enhanced reactivations can preserve behavioral improvements in learning, though their neurophysiological effects remain inconclusive.

## 1 INTRODUCTION

It is generally accepted that motor skill learning has two phases. The initial, or fast learning phase typically refers to improvements that occur within a single session of practice, at least when learning a relatively simple motor task (Dayan & Cohen, 2011; Karni et al., 1995). The second, slow learning phase occurs over a much longer period of time and is arguably indefinite, assuming the motor skill continues to be practiced (Doyon & Benali, 2005; Fischer et al., 2005; Walker et al., 2002). In terms of embedding or acquiring a motor skill and becoming proficient at it, most of the focus is on the brain regions, substrates, consolidation and practice frequency/duration of the slow learning phase (Dayan & Cohen, 2011; Ungerleider et al., 2002).

The slow learning phase has informally been popularized by Malcolm Gladwell in his book Outliers Gladwell (2008), and based on the original study by Ericsson et al. (1993), where a time duration was assigned to designate when ‘expertise’ is achieved. While expertise is on the opposite end of the spectrum from initially learning a new skill, the part that is the most important is what happens in between these two periods. The period of days, weeks, and months of practice of a new motor skill depend largely on the amount of practice within a single session and what that practice is focused on (Christiansen et al., 2020; Savion‐Lemieux & Penhune, 2005; Walker et al., 2002; Yamada et al., 2019). For instance, if the practice is deliberate, meaning it is purposeful and structured, and lacking any interference that could take away or divert attention (Memmert, 2011), then the process of learning should be hastened.

The acquisition of a new motor skill requires the formation of new motor memories, primarily through consolidation (Doyon & Benali, 2005; Fischer et al., 2005; Muellbacher et al., 2002). Consolidation makes the motor memory more established and resistant to change while improving performance by increasing task speed and reducing variability (Shmuelof et al., 2012). Both sleep and the passage of time are critical to the consolidating the motor memories (de Beukelaar et al., 2016; Fischer et al., 2002; Walker et al., 2002). However, once the motor memory is consolidated and robust, it brings into question the necessity of frequent, long‐duration practices to retain that skill. For example, Herszage et al. (2021) used very brief memory reactivations of a keyboard button‐pressing sequence task and found that subjects performed just as well as those that had practiced with many trials of the same task. Corticospinal excitability, tested with transcranial magnetic stimulation over the primary motor cortex (M1), correlated with retest performance, showing that subjects learning the task with reactivation had the most M1 plasticity (Herszage et al., 2021). This novel study shows a scenario where once a motor memory is fully consolidated, the level of practice needed to continue to learn and improve is greatly reduced.

Another tool that has been used to aid in motor skill learning is transcranial direct current stimulation (tDCS). tDCS can change neuronal excitability by altering resting membrane potential without suprathreshold activation of the targeted brain areas, thereby increasing the likelihood of the necessary groups of neurons firing together (Meek et al., 2021; Nitsche et al., 2003; Reis & Fritsch, 2011; Wiethoff et al., 2014). This can be driven by changes in the GABAergic function of the cells (Fritsch et al., 2010), long‐term potentiation mechanisms (Spampinato & Celnik, 2017), or even the indirect influence of glial cells (Gellner et al., 2016). Regardless of the exact mechanism, it is well established that M1 tDCS can enhance finger and hand dexterity movements, such as sequential finger tapping tasks and sequential visual isometric pinch force tasks (Buch et al., 2017). Additionally, tDCS of M1 has been shown to aid in consolidation of new motor skills (Kim et al., 2021).

The purpose of the present study was to examine the effect of brief reactivations on learning of a Choice Response Task (CRT) and to determine if applying tDCS to M1 can further accelerate learning or at least enable the lesser practice from the brief reactivations to improve as much as groups that are allowed full practice. This is a progression from the study by Herszage et al. (2021) where they were using a repetitive sequence of button pressing that did not change. Our CRT was unpredictable, but also relatively easy to learn. A secondary aim of this study is to investigate differences in sexes in a reaction time task, where there is known to be a paradox between females interpreting the correct answer faster, but males having quicker motor movement (Blough & Slavin, 1987; Landauer et al., 1980). Ultimately, the goal is to determine if motor skill learning can be achieved with less overall time required for practice, which has carry‐over to rehabilitation settings where practice usually requires trained supervision and significant resources.

## 2 METHODS

### 2.1 Participants

A total of 120 healthy young adults (62 males and 58 females), between the ages of 18 and 45, participated in the study. Pilot data collected showed mean reaction times of 350–450 ms with pooled standard deviations of 35, which yields a Cohen's *f* of 0.976. Across 6 groups, this required 20 subjects per group to achieve a power of 0.8 with a significance level of 0.05. Participants reported no brain injury, neurological disorder, or skeletal muscle disorder in their upper extremities and were physically capable of performing the task. Subjects were instructed to avoid any over‐the‐counter stimulants containing caffeine (coffee, soda, tea, energy drinks, pre‐workout supplements) or nicotine (tobacco, nicotine pouches, gums, etc.) for at least 12 h before their first and last visits. At the beginning of the first visit, subjects were given a copy of the informed consent, which was explained to them by the researchers. All subjects then provided written consent. They were also given a brain stimulation screening questionnaire and had their handedness determined using the Edinburgh Handedness Inventory (Oldfield, 1971). The study was approved by the Indiana University Institutional Review Board (#15058) and was conducted according to the Declaration of Helsinki.

### 2.2 Experimental design

**FIGURE 1:**
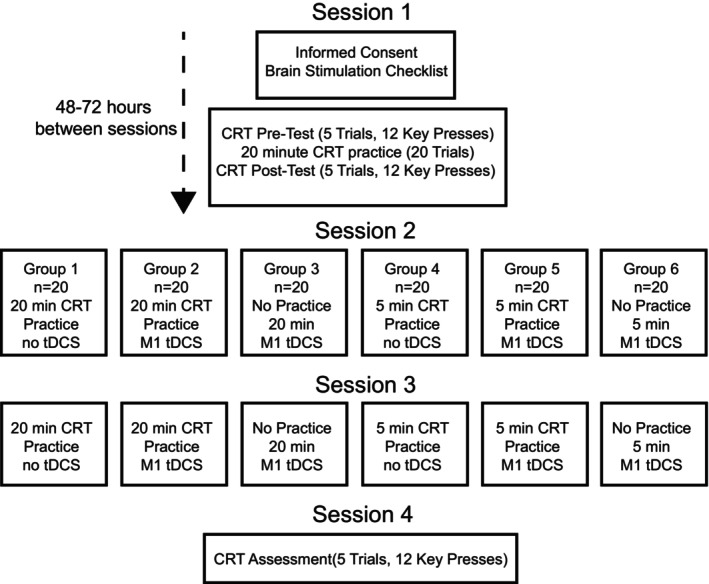
Displays the experimental procedures and the different groups used in the study.

The study utilized a randomized, single‐blinded, between‐subjects study design. All subjects visited the lab for 4 testing sessions. The first and last sessions were the same for everyone. After filling out the forms in the first session, the subjects completed a familiarization trial of 12 key presses of a choice reaction task (CRT, 1 trial = 12 key presses). After a short rest, they completed 5 trials, with 30s between trials as the pre‐test. In that same session, they did a 20‐min practice session with 20 trials. Then, after another short rest, they completed 5 more trials for the post‐test. The fourth session just involved coming into the lab for a follow‐up, completing 5 more trials. The requirements for sessions 2 and 3 depended on what group they were randomized to. Subjects had 48–72 h between sessions to account for weekends (Figure 1).

Subjects were randomized into one of six experimental groups that determined what they did in sessions 2 and 3: (1) 20 min of practice with the CRT with no tDCS, (2) 20 min of practice with the CRT with tDCS over M1, (3) 20 min of tDCS only with no practice of the CRT, (4) 5 min of practice with the CRT with no tDCS, (5) 5 min of practice with the CRT with tDCS of M1, and (6) 5 min of tDCS only with no practice of the CRT. Subjects only performed the tasks listed for their group in sessions 2 and 3.

### 2.3 Choice reaction time task (CRT)

**FIGURE 2:**
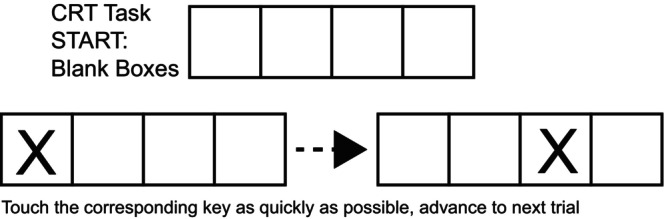
Shows an example of what the CRT task looks like to the subject.

A choice reaction time task was used based on the original Deary‐Liewald choice reaction task (Deary et al., 2011) from open‐source software (Stoet, 2010, 2017). Briefly, subjects were presented with four blank boxes and when an X appeared in one of the four boxes the participant selected the corresponding key that represented the box that displays the X. The participants used the four fingers of their non‐dominant hand to press the keys. Subjects were instructed to focus on the computer screen and not interact with the investigators during the trials. Each trial block consisted of 12 key presses that took about 30 s to complete and then subjects were always given 30 s of rest between trials. The output of the task measured the time it took to press the correct button (ms) and the accuracy of pressing the correct button (Figure 2).

### 2.4 tDCS stimulation

tDCS was delivered via a Soterix Medical 1 × 1 low‐intensity transcranial direct current stimulators during session 2 and 3 for groups 2, 3, 5, and 6. The stimulation parameters used in this study have been established as safe and effective in previous literature (Lefaucheur et al., 2017). No adverse effects were observed or reported in any of the subjects in the tDCS stimulation experimental groups in the present study. A pair of rubber electrodes (5 × 5 cm in size) were placed inside of saline soaked sponges and each were fixed on the scalp with a rubber strap. The active electrode was placed on the hand region of M1 (C3 and C4 according to 10–20 EEG Coordinate System, depending on the non‐dominant M1), while the reference electrode was placed on the ipsilateral scalp in the supraorbital region (Fp1 or Fp2, respectively). Anodal tDCS was delivered to M1 with a current intensity of 2 mA and for durations of 20‐min (groups 2 and 3) or 5‐min (groups 5 and 6). Subjects in the groups that did not receive active tDCS (Groups 1 and 4), were subject to a SHAM protocol that has been shown to elicit the sensation of tDCS, but without the physiological effects (Gandiga et al., 2006). Briefly, there was a 30 s ramp up to 2 mA immediately followed by a 30 s ramp down at the beginning and again at the end of each session.

### 2.5 Data analysis

CRT data was separated into reaction times (RTs) and errors (wrong button pressing) for pre‐ and post‐testing within session 1 and for comparisons of post‐testing in session 1 to testing in session 4. For the purposes of this study, early learning will refer to the changes in the CRT within the first session, and late learning will refer to the changes between sessions 1 and 4. RTs and errors were averaged across the 12 key presses within each trial and then across the five trials. The average fastest visual reaction times are ~220 ms, so any trials shorter than that were discarded from the analysis (Jain et al., 2015; Welford et al., 1980). Similarly, we discarded any reaction times that were longer than the overall group mean plus 3 standard deviations. Out of the entire study, only 3 trials were discarded based on overly long reaction times.

### 2.6 Statistics

A one‐way ANOVA was used to compare the initial RTs across the 6 groups to ensure they were similar at the beginning of the experiment. A mixed model three‐way ANOVA was used to analyze RTs and the number of errors made, with time being the within‐subjects factor (session 1 pre‐test, session 1 post‐test, and session 4 post‐test) and group (6 groups) and sex (2 sexes) being the between‐subject factors.

## 3 RESULTS

**FIGURE 3:**
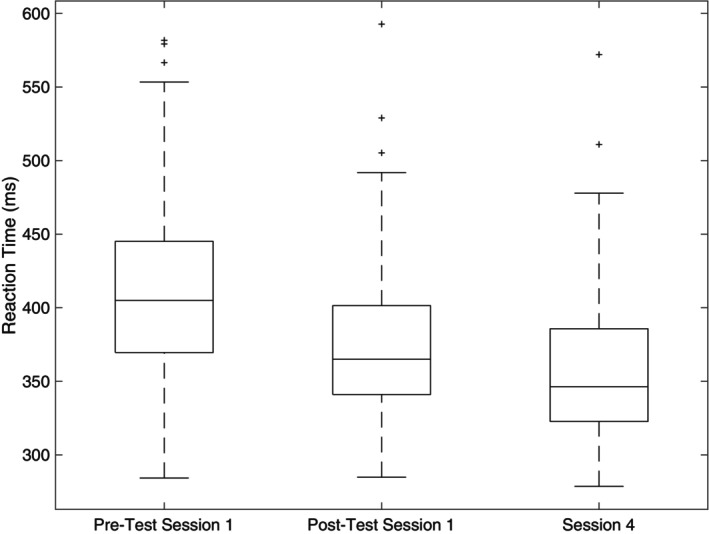
Boxplot displaying reaction times from session 1 pre and post and session 4 collapsed across all subjects. Box includes median line and 25th and 75th percentiles. Whiskers denote maximum and minimum data points, and + symbols represent outliers. These outliers did not meet the original definition of mean ± 3 standard deviations that we used when scrubbing the original dataset, so they were included.

There were no differences between groups in either RT (*p* = 0.834), or error (*p* = 0.977) in the pre‐testing of session 1, indicating the groups were similar. The results of the mixed model three‐way ANOVA showed a significant main effect for time (*F*(2, 118) = 18.592, *p* < 0.001, *η*_p_^2^ = 0.376) for the RT measure (Figure 3). Specifically, subjects improved their RT from the pre‐test of session 1 from 423.5 ± 116.8 ms to 357.9 ± 63.4 ms at the post‐test of session 1 (*p* < 0.001). The follow‐up testing in session 4 resulted in RTs of 377.8 ± 66.2 ms, which were not different from the post‐test of session 1 (*p* = 0.172) but were still significantly shorter than the pre‐test of session 1 (*p* < 0.001).

**FIGURE 4:**
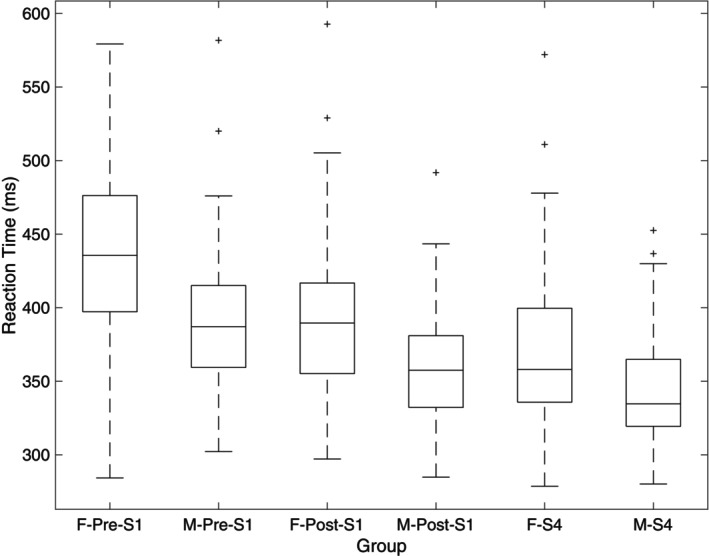
Boxplot displaying reaction times between males and females from session 1 pre and post and session 4. Box includes median line and 25th and 75th percentiles. Whiskers denote maximum and minimum data points, and + symbols represent outliers. These outliers did not meet the original definition of mean ± 3 standard deviations that we used when scrubbing the original dataset, so they were included.

**FIGURE 5:**
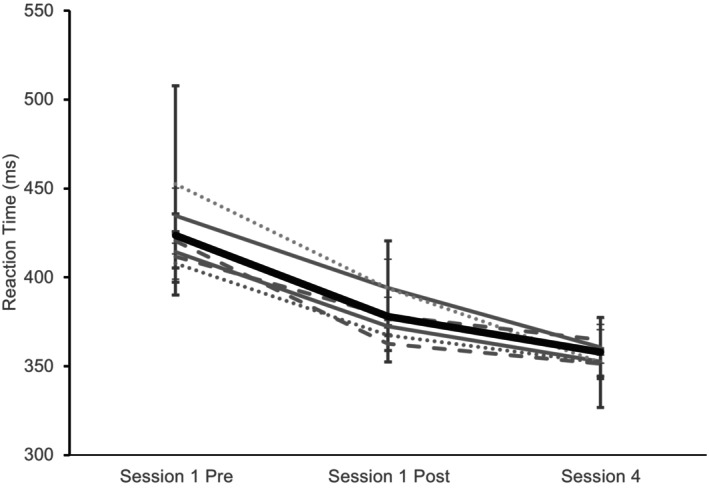
Group reaction times from session 1 pre and post and session 4. The thin, gray solid lines are groups 1 and 2 that had full practice. The thin, gray dashed lines are groups 4 and 5 that had 5‐min reactivations. The dotted lines are tDCS only (groups 3 and 6) that did not have practice. The thick black line is the average collapsed across all subjects.

There was a between‐subjects effect of sex on RT (*F*(1, 118) = 8.468, *p* = 0.004, η_p_^2^ = 0.073), as females were slower overall in the RT task. Additionally, there was a time × sex interaction for RT (*F*(1, 118) = 5.574, *p* = 0.02, η_p_^2^ = 0.049; Figure 4). There was no significant difference between subjects' effect on RT for group (*p* = 0.759, η_p_^2^ = 0.024), and there were no significant interactions for time × group (*p* = 0.976, η_p_^2^ = 0.012; Figure 5), group × sex (*p* = 0.942, η_p_^2^ = 0.009), or time × group × sex (*p* = 0.329, η_p_^2^ = 0.035). For the number of errors committed in the task, there was a trend for a significant effect of time (*F*(2, 118) = 2.876, *p* = 0.064, η_p_^2^ = 0.031). However, there were no effects of sex or group, and there were no significant interactions between these variables (*p* = 0.160–0.685).

## 4 DISCUSSION

This study sought to examine whether brief reactivations of a motor task (CRT), when paired with anodal tDCS (a‐tDCS) over the primary motor cortex (M1), could further accelerate motor learning, or at least allow less practice from brief reactivations to improve as much as groups who had full practice. Our findings add to the growing body of literature that investigates the underlying mechanisms of the phases of learning, the application and effectiveness of a‐tDCS and the potential differences in behavioral changes between the sexes (Blough & Slavin, 1987; Herszage et al., 2021; Landauer et al., 1980).

The behavioral findings of our study showed that reaction time (RT) improved significantly over time, suggesting the presence of task‐related learning or adaptation effects. For all experimental groups, the RTs improved from the pre‐test to post‐test in session 1, and although some loss of performance was observed by session 4, RTs remained significantly faster than baseline. Our results are consistent with the occurrence of the fast phase of motor learning. During this phase, rapid performance gains are seen within a single session due to neural adaptations that occur primarily within the primary motor cortex (M1). It is believed that during this phase synaptic modifications such as long‐term potentiation (LTP) and long‐term depression (LTD) facilitate early neuroplastic changes that are required for the acquisition of a new motor skill (Dayan & Cohen, 2011; Karni et al., 1995; Ziemann, 2004).

Although some performance decline was seen by session 4, RTs remained significantly better than at baseline, suggesting that the gains made during the fast‐learning phase were mostly retained. Retention of reaction times supports the occurrence of offline consolidation. When we progress from fast to slow motor learning we rely on the consolidation of that motor task, where that motor memory becomes more stable and less likely to be affected by external interference, which is typically referred to as offline motor learning (Doyon & Benali, 2005). It is important to understand that previously consolidated memories are not immune to further modifications, reactivation of a consolidated memory can make it susceptible to interference or enhancement (Nader et al., 2000; Walker et al., 2002). After motor skills are acquired and consolidated, they can be retained over extended periods of time or forgotten. In our study we showed that under specific laboratory settings, retention of motor skills lasted for at least 2 weeks. The CRT task that we utilized did not have a specific sequence, and therefore would be considered random practice, we chose this type of practice due to previous research showing that random practice can lead to more rapid memory stabilization (Tanaka et al., 2010). This is in line and has been well documented in other research showing long‐term retention even with short practice sessions, indicating that long‐term retention is strongly dependent on successful consolidation (Abe et al., 2011; Savion‐Lemieux & Penhune, 2005). All of this taken together, we can conclude that although the reactivation sessions between day 1 and 4 may not have improved reaction time more than baseline improvements, they did stabilize and retain the motor improvements from the first to the last session. This indicates that the reconsolidation that occurred during sessions 2 and 3 positively influenced motor performance by stabilizing and reconsolidating the motor memory. This finding compliments the findings by Herszage et al., who found that short‐reactivations of a motor memory was sufficient enough enhance motor performance (2021).

An alternative explanation that cannot be ruled out based on the current data is that the task was so easy to learn, that exposure to it in a single session is enough to become proficient at it. The lack of a difference in reaction time between the group that just had 5 min of tDCS with no practice, which is basically a control group, and the groups that had full practice, would suggest that the task was already learned. The CRT task we used may not be complex enough to require any practice, let alone reactivations or full length practice sessions, whereas with the task used by Herszage et al. (2021) there were improvements in both practice groups beyond what was observed in the control group.

Despite previous work showing that tDCS can modulate motor learning, our results did not provide statistically significant differences in CRT performance between the experimental groups. This finding warrants careful consideration, as previous research has shown that a‐tDCS applied over the M1 can enhance synaptic plasticity and facilitate motor acquisition (Nitsche et al., 2003; Reis & Fritsch, 2011). tDCS has been suggested to strengthen motor memory consolidation, specifically, when paired with motor practice. This is thought to be due to the increase in cortical excitability and support of LTP like mechanisms (Stagg & Nitsche, 2011). It has also been shown to reduce variability in performance and accelerate learning in simple and complex motor tasks. Therefore, our expectation was that applying tDCS in combination with either full practice or brief reactivations would yield higher behavioral outcomes compared to the non‐tDCS groups. The absence of significant group‐level effects may reflect several contributing factors that have been noted in previous literature such as inter‐individual variability responsiveness to tDCS, anatomical and functional differences in cortical target areas, state‐dependent effects influenced by prior task engagement, or ceiling effects of the task due to the simplicity of it (Furuya et al., 2014; Horvath et al., 2015; Perez et al., 2025; Rosen et al., 2016; Vergallito et al., 2022).

These findings lead to the conclusion that the effects of tDCS can be small and highly variable across individuals (Horvath et al., 2015; Prehn & Floel, 2015). Although our study maintained a high level of protocol consistency, including the same CRT task, environment, and lack of distractions, uncontrollable participant level differences may have contributed to the outcome. These include variability in cognitive strategies, attentional engagement, and motivation. Even small lapses in attention or inconsistent engagement can significantly influence reaction time, adding noise that could mask true group‐level effects (Falcone et al., 2018). Therefore, the lack of findings does not necessarily indicate a lack of efficacy for tDCS but instead, emphasize the complexity and variability of its neuromodulatory impact.

With regards to brief reactivation, although there were no statistically significant differences across groups, we did see that participants in the brief reactivation group that received tDCS (group 5) achieved performance improvements comparable to those who were in the full practice group, despite spending considerably less time practicing. This finding, while still novel and exploratory, is comparable to the conclusions drawn by Herszage et al. (2021), who demonstrated that well‐timed, error free reactivations could stabilize and enhance motor memories by enhancing the reconsolidation process. Overall, these findings suggest the potential for brief reactivation combined with neuromodulation to serve as an efficient alternative to prolonged practice, specifically when the time to train is constrained. These observations should encourage re‐examination of assumptions about practice dosage, as growing evidence supports that motor learning may not always require extensive repetition, especially when motor memories are actively re‐engaged and stabilized through neuroplastic interventions (Vergallito et al., 2022).

Sex was found to be a significant between‐subjects factor, with females demonstrating overall slower RTs across sessions compared to males. Moreover, the presence of a time × sex interaction suggests that the pattern of RT improvement may differ slightly by sex. This finding aligns with existing literature indicating that males tend to respond faster than females in reaction time‐type tasks (Bianco et al., 2020; Blough & Slavin, 1987; Landauer et al., 1980). Researchers have suggested that this difference arises from the pattern of cognitive processing, where males rely more on reactive, speed‐oriented strategies and females engage in more proactive and cautious processing (Bianco et al., 2020; Blough & Slavin, 1987). Neurophysiological studies have also supported this through electroencephalography (EEG) recordings that found that females had more event‐related potential (ERP) data showing larger prefrontal negativity and visual negativity prior to the onset of a stimulus, which indicate an increase in cognitive and sensory preparation. Males demonstrate larger readiness potential data indicating enhanced motor readiness and preference for rapid response execution (Bianco et al., 2020; Di Russo et al., 2016). Given the potential differences in cognitive processing between sexes, it may be expected that while males had faster RTs, they also may be more prone to errors. We did not observe sex differences in the number of errors, though error rates in the task we used were very low for everyone. Choosing a task that is more difficult may have been able to demonstrate the sex differences between speed and error more appropriately with what has been discussed in the literature. Taken together, our data along with previous research supports that RT is not simply behavioral but are possibly rooted in cognitive styles and neural processing patterns.

## 5 CONCLUSION

From a larger perspective, this study contributes to the evolving literature on training paradigms for motor learning. While the promise of tDCS remains strong, especially in its potential application to populations requiring rapid skill acquisition such as athletes, surgeons, and individuals undergoing rehabilitation, the current findings emphasize the importance of refining stimulation protocols, accounting for individual variability, and using larger samples to detect potentially subtle neuromodulatory effects.

Future studies should prioritize adaptive stimulation models, including personalized dosing strategies, neuroimaging‐guided or electrical field modeling of electrode placement, and incorporation of state‐dependent measures to better understand when and for whom tDCS may be most effective. Additionally, further exploration into the neural correlates of brief reactivations, especially under tightly controlled behavioral and physiological conditions, may help uncover whether these sessions can reliably serve as a time‐efficient alternative to traditional, prolonged practice. If brief reactivations continue to be shown effective, this can significantly lessen the time and resources needed in clinical settings and other applications, reducing the potential for excessive fatigue or overuse. In conclusion, while brief motor practice leads to measurable improvements in task performance, the application of tDCS under the current study parameters did not enhance these gains. These findings highlight both the potential and limitations of tDCS in applied motor learning settings and underscore the continued need for rigorous, targeted investigation into the interplay between brain stimulation and motor memory.

## AUTHOR CONTRIBUTIONS

Michaela A. Wilson, Brach Poston, and Zachary A. Riley contributed to the design and implementation of the research, data collection, analysis of the results, and to the writing of the manuscript.

## FUNDING INFORMATION

No funding was received for this work.

## CONFLICT OF INTEREST STATEMENT

None of the authors have any conflicts of interests.

## ETHICS STATEMENT

I testify on behalf of all co‐authors that our article submitted followed ethical principles in publishing.

## Data Availability

Data is available upon request.
